# High incidence of adverse events during intra-hospital transport of critically ill patients and new related risk factors: a prospective, multicenter study in China

**DOI:** 10.1186/s13054-016-1183-y

**Published:** 2016-01-18

**Authors:** Liu Jia, Hongliang Wang, Yang Gao, Haitao Liu, Kaijiang Yu

**Affiliations:** 1 Department of Critical Care Medicine, the Second Affiliated Hospital of Harbin Medical University, Harbin, Heilongjiang China; 2 Department of Critical Care Medicine, the Third Affiliated Hospital of Harbin Medical University, Harbin, Heilongjiang China

## Abstract

**Background:**

The aim of the present study was to investigate the incidence of adverse events (AEs) during intra-hospital transport (IHT) of critically ill patients and evaluate the risk factors associated with these events.

**Methods:**

This prospective multicenter observational study was performed in 34 intensive care units in China during 20 consecutive days from 5 November to 25 November 2012. All consecutive patients who required IHT for diagnostic testing or therapeutic procedures during the study period were included. All AEs that occurred during IHT were recorded. The incidence of AEs was defined as the rate of transports with at least one AE. The statistical analysis included a description of demographic and clinical characteristics of the cohort as well as identification of risk factors for AEs during IHT by univariate and multivariate logistic regression analyses.

**Results:**

In total, 441 IHTs of 369 critically ill patients were analyzed. The overall incidence of AEs was 79.8 % (352 IHTs). The proportion of equipment- and staff-related adverse events was 7.9 % (35 IHTs). The rate of patient-related adverse events (P-AEs) was 79.4 % (349 IHTs). The rates of vital sign–related P-AEs and arterial blood gas analysis–related P-AEs were 57.1 % (252 IHTs) and 46.9 % (207 IHTs), respectively. The incidence of critical P-AEs was 33.1 % (146 IHTs). The rates of vital sign–related critical P-AEs and arterial blood gas analysis–related critical P-AEs were 22.9 % (101 IHTs) and 15.0 % (66 IHTs), respectively. All data collected in our study were considered potential risk factors. In the multivariate analysis, predictive factors for P-AEs were pH, partial pressure of carbon dioxide in arterial blood, lactate level, glucose level, and heart rate before IHT. Furthermore, the Acute Physiology and Chronic Health Evaluation II score, partial pressure of oxygen in arterial blood, lactate level, glucose level, heart rate, respiratory rate, pulse oximetry, and sedation before transport were independent influential factors for critical P-AEs during IHT.

**Conclusions:**

The incidence of P-AEs during IHT of critically ill patients was high. Risk factors for P-AEs during IHT were identified. Strategies are needed to reduce their frequency.

**Trial registration:**

Chinese Clinical Trial Register identifier ChiCTR-OCS-12002661. Registered 5 November 2012.

## Background

Intra-hospital transport (IHT) is an inevitable and important part of intensive care unit (ICU) care. IHT is frequently required to perform diagnostic or therapeutic procedures for critically ill patients. Transported patients have more significant illnesses than patients not requiring transport [1]. Additionally, adverse events (AEs) during IHT occur commonly, and transported patients have significantly higher risks than non-transported patients in the ICU [1–3]. The decision to transport a critically ill patient is based on an assessment of the potential benefits and risks [4]. Knowledge of the incidence of AEs and risk factors for AEs during IHT is essential for scheduling safe ICU patient transport.

The overall incidence of AEs during IHT of critically ill patients reportedly ranges from 1.7 % to 75.7 % [5, 6]. Several explanations have been proposed for this wide range. One explanation is the different types of patients studied. Patients studied include those in the medical ICU [3], surgical ICU [7], anesthesiological ICU [6], neurological ICU [8], and emergency department [9–11]; mechanically ventilated patients [2, 3, 12]; and patients going to different transport destinations. Another explanation is the different definitions of AEs used among various reports. The most commonly used AE classifications are equipment/staff- and patient-related adverse events (P-AE) [12–14]. However, there is no standard definition of respiratory or circulatory AEs. A third explanation for the wide range of AEs during IHT is the different time periods studied. AEs related to IHT can occur during transport or secondarily even on the following day. Finally, the wide range may be explained by different programs used to limit AEs. This includes the use of specialized transport teams during IHT [5] or the use of designed transport checklists by acting nurses before the patients are transported [10].

To our knowledge, this is the first multicenter observational study to comprehensively identify the incidence and risk factors of AEs during the IHT of different ICU patients. These findings will help train a cadre of IHT personnel to perform safer ICU patient transport.

## Methods

### Study design and patients

**Fig. 1:**
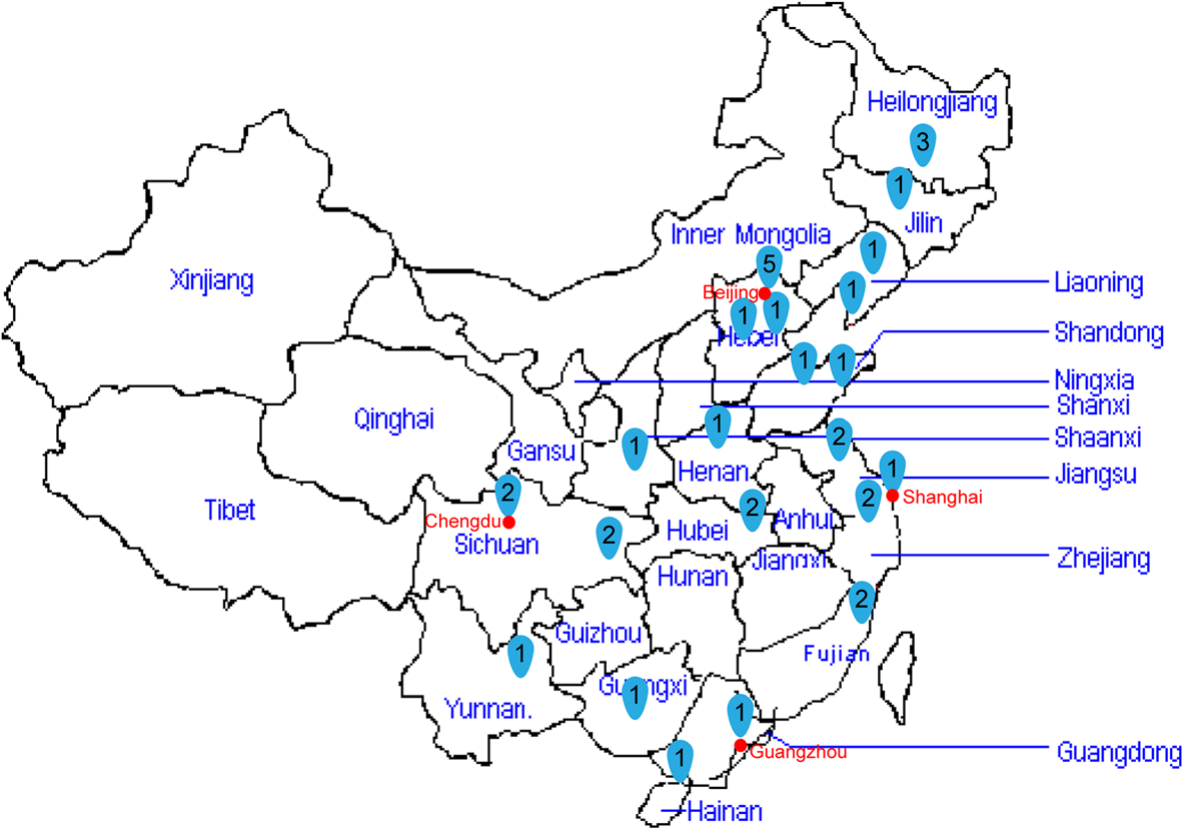
Thirty-four clinical centers in China participated in this study. Numbers represent number of centers at each location

A prospective multicenter observational study was carried out in 34 closed ICUs (with staff members formally trained in critical care) in China during 20 consecutive days from 5 November to 25 November 2012 (Fig. 1). All consecutive patients who required IHT for diagnostic testing or therapeutic procedures during the study period were included. The study design and informed consent form were both approved by the medical ethics committee of the Second Affiliated Hospital of Harbin Medical University (the organizer institution). The study was registered in the Chinese Clinical Trial Register as ChiCTR-OCS-12002661.

No specific protocol, including special staff training, was used to manage critically ill patients before or during IHT. The risk or benefit of IHT for critically ill patients was assessed by the in-charge ICU physician. Patients who were transported to the operating room or the general ward after diagnostic testing were excluded. Written informed consent was obtained from the patients or their guardians or family members.

### Data collection and outcome measures

Each participating ICU had a written procedure for data collection. All data were collected by trained observers from each participating ICU using a case report form. The observation period was divided into three parts: pre-IHT, IHT, and post-IHT. The pre-IHT period was defined as the time before the patient departed for IHT, and the post-IHT period was defined as the time after the patient returned to their ICU bed. Each period was measured with a maximum error of 5 minutes. All data in the pre-IHT period was established as baseline information.

**Fig. 2:**
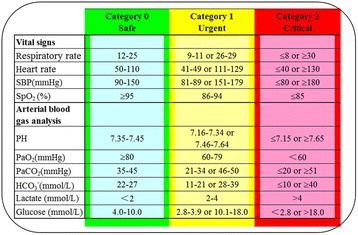
Severity categories of vital signs and arterial blood gas analysis. *SBP* systolic blood pressure, *SpO*_*2*_ pulse oximetry, *PaO*_*2*_ partial pressure of oxygen in arterial blood, *PaCO*_*2*_ partial pressure of carbon dioxide in arterial blood, *HCO*_*3*_^*−*^ bicarbonate level

Patient characteristics were collected immediately after the patient was enrolled in the study. The severity of illness was determined using Acute Physiology and Chronic Health Evaluation (APACHE) II scores obtained on the day of transport. The Glasgow Coma Scale (GCS) score was evaluated on the basis of the patient’s last condition before transport if the patient had been sedated. IHT characteristics such as the transport destination were also recorded. Arterial blood gas analysis findings [pH, partial pressure of oxygen in arterial blood (PaO_2_), partial pressure of carbon dioxide in arterial blood (PaCO_2_), bicarbonate, lactate level, and glucose level] were reviewed during the pre-IHT and post-IHT periods. Transport monitors were used to collect vital signs [systolic blood pressure, diastolic blood pressure, mean arterial pressure, heart rate (HR), respiratory rate (RR), and pulse oximetry (SpO_2_)] every 5 minutes during the entire observation period. The vital signs and arterial blood gas analysis findings were categorized according to severity [15–17] (Fig. 2).

All AEs that occurred during IHT were recorded, regardless of whether a treatment was performed. AEs were classified as equipment- and staff-related adverse events (E-AEs) or as P-AEs. A P-AE was defined as any event that affected patient stability. A vital sign–related or arterial blood gas analysis–related P-AE was defined as an AE associated with detection of abnormal or more severe monitored parameters during the IHT period and the post-IHT period. A critical P-AE was defined as a vital sign or arterial blood gas analysis parameter with more severe abnormality detected (category 2 or worse), as well as other life-threatening events such as airway obstruction, accidental extubation, cardiac arrest, malignant arrhythmias, and others. Different AEs might occur during one transport. The incidence of AEs was defined as the rate of transports with at least one AE.

### Data analysis

The sample size was calculated using the formula *n* = *z* ^2^_α/2_π(1 − π)/δ^2^ (where α = 0.05, *z*_α/2_ = 1.96, π = 50 %, and δ = 5 %). Because the known incidence of AEs from previous studies of ICU patients during transport varies from 1.7 % to 75.7 %, we calculated the sample size as 384 IHTs with the assumption that 50 % of patients would experience an AE. We anticipated that 10 % of the data would be missing, which increased the target IHT sample size to 422.

Statistical analysis was performed using SAS software (release 9.13, serial 989155; SAS Institute, Cary, NC, USA). Quantitative variables are reported as mean with standard deviation or as median with 25th and 75th percentiles. Qualitative data are described as values or percentages. A *P* value <0.05 was considered statistically significant.

Possible risk factors for P-AEs during IHT were identified first by univariate logistic regression analysis. Those with a significance level <0.05 were included in a stepwise multivariate logistic regression analysis. Results are reported as the odds ratio (OR) with 95 % confidence interval (CI).

## Results

### Patients and IHTs

**Fig. 3:**
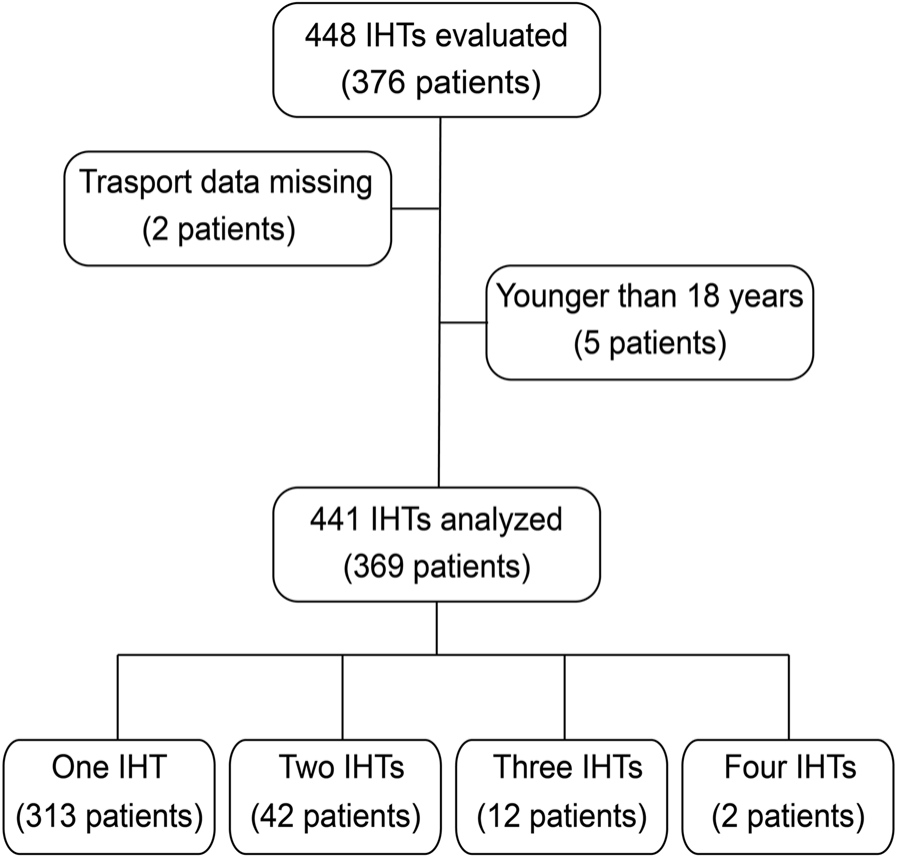
Patients undergoing intra-hospital (IHT) transport

In total, 376 critically ill patients were enrolled during the study period (Fig. 3). These patients underwent 448 IHTs. Seven IHTs were excluded because of a lack of data documentation (*n* = 2) or a patient age <18 years (*n* = 5). Thus, 369 critically ill patients with 441 IHTs were included in the analysis. Fifty-six patients underwent more than 1 IHT, including 42 with 2 IHTs, 12 with 3 IHTs, and 2 with 4 IHTs. Thus, 72 IHTs were not the first IHT for that patient during that ICU stay. Patient and clinical characteristics before IHT were determined (Tables 1 and 2).

**Table 1:** Patient characteristics (*N* = 441 intra-hospital transports evaluated)

Patient characteristic	Median	[25th–75th percentile]	Mean ± SD	Transports (*n*)	Transports (%)
Age, yr	60	[46–72]	58.8 ± 18.0		
Sex					
Male				288	65.3
Female				153	34.7
Weight, kg	65	[58–70]	65.6 ± 12.5		
ICU admission type					
Medical				163	37.0
Surgical				195	44.2
Trauma				46	10.4
Other				37	8.4
APACHE II score	14	[9–21]	15.4 ± 8.1		
GCS score	15	[9–15]	11.9 ± 4.2		

*APACHE* Acute Physiology and Chronic Health Evaluation, *GCS* Glasgow Coma Scale, *ICU* intensive care unit *SD* standard deviation

**Table 2:** Clinical characteristics before IHT (*N* = 441 intra-hospital transports evaluated)

Clinical characteristic	Transports (*n*)	Transports (%)
Artificial airway	255	57.8
Intubation	144	32.7
Tracheostomy	111	25.2
Mechanical ventilation		
No ventilatory support	237	53.7
Nasal catheter	155	35.1
Face mask	82	18.6
Ventilatory support	204	46.3
Non-invasive ventilation	8	1.8
Invasive ventilation	196	44.4
Catheter use		
Central venous	317	71.9
Peripheral vein	171	38.8
Arterial	107	24.3
Nasogastric tube	291	66.0
Foley catheter	311	70.5
Drainage catheter	138	31.3
Other	22	5.0
Vasoactive drug support^a^	107	24.3
Catecholamines	62	14.1
Vasodilator	28	6.4
Other	10	2.3
More than one of above	7	1.6
Sedation^a^	102	23.1
Midazolam	36	8.2
Propofol	35	7.9
Dexmedetomidine	18	4.1
Other	3	0.7
More than one of the above	10	2.3
Analgesia^a^	67	15.2
Opioid	60	13.6
Non-opioid	7	1.6
Severity categories of vital signs^b^		
Respiratory rate		
Category 0	346	78.5
Category 1	48	10.9
Category 2	47	10.7
Heart rate		
Category 0	355	80.5
Category 1	65	14.7
Category 2	21	4.8
Systolic blood pressure		
Category 0	381	86.4
Category 1	51	11.6
Category 2	9	2.0
SpO_2_		
Category 0	391	88.7
Category 1	47	10.7
Category 2	3	0.7
Severity categories of ABG^b^		
pH		
Category 0	253	57.4
Category 1	186	42.2
Category 2	2	0.5
PaO_2_		
Category 0	326	73.9
Category 1	95	21.5
Category 2	20	4.5
PaCO_2_		
Category 0	197	44.7
Category 1	218	49.4
Category 2	26	5.9
Bicarbonate level		
Category 0	220	49.9
Category 1	217	49.2
Category 2	4	0.9
Lactate level		
Category 0	345	78.2
Category 1	72	16.3
Category 2	24	5.4
Glucose level		
Category 0	333	75.5
Category 1	101	22.9
Category 2	7	1.6

*SBP* systolic blood pressure, *SpO*_*2*_ pulse oximetry, *ABG* arterial blood gas, *PaO*_*2*_ partial pressure of oxygen in arterial blood, *PaCO*_*2*_ partial pressure of carbon dioxide in arterial blood

^a^Medications were delivered as continuous infusions

^b^Severity categories defined in Fig. 2

Patient IHT characteristics were evaluated (Table 3). In total, 433 IHTs (98.2 %) were to only one location, most commonly for computed tomographic imaging (380 IHTs, 86.2 %). Other destinations included ultrasonography (18 IHTs, 4.1 %), radiation treatment (8 IHTs, 1.8 %), magnetic resonance imaging (7 IHTs, 1.6 %), endoscopy (4 IHTs, 0.9 %), and angiography (4 IHTs, 0.9 %). Only eight IHTs (1.8 %) were to more than one location. Eighty-three IHTs (18.8 %) were emergent. The majority of IHTs (438 IHTs, 99.3 %) were carried out successfully. Three patients were not transferred successfully, owing to serious AEs (airway obstruction, accidental extubation, and a low SpO_2_ of 60 %, respectively). A minority of IHTs were performed between 5:00 pm and 8 am (27 IHTs, 6.1 %). The median duration of IHT was 25 minutes (25th–75th percentile range 20–35 minutes).

**Table 3:** Intra-hospital transport characteristics (*N* = 441 intra-hospital transports evaluated)

IHT characteristic	Transports (*n*)	Transports (%)
Transport destination		
Computed tomography	380	86.2
Ultrasonography	18	4.1
Radiation therapy	8	1.8
Magnetic resonance imaging	7	1.6
Digestive endoscopy	4	0.9
Angiography	4	0.9
Other	12	2.7
Multiple destinations	8	1.8
Transport type		
Emergency	83	18.8
Elective	358	81.2
Multiple IHTs of one patient	72	16.3
Transport time		
Daytime (8:00 am–5:00 pm)	414	93.9
Nighttime (5:00 pm–8:00 am)	27	6.1
Medications administered during IHT^a^		
Analgesia	45	10.2
Sedation	80	18.1
Vasoactive drug support	92	20.7
Completed transports	438	99.3

*IHT* intra-hospital transport

^a^Medications were delivered as continuous infusion

### AEs during IHT

Table 4 shows the incidence and types of AEs during IHT. The overall incidence of AEs was 79.8 % (352 IHTs). The proportion of E-AEs was 7.9 % (35 IHTs). Most of these involved a disconnected monitor power source or monitor power failure (11 IHTs, 2.5 %), disconnected or depleted oxygen supply (9 IHTs, 2.0 %), or unexpected delays during transport (8 IHTs, 1.8 %). The rate of P-AEs was 79.4 % (349 IHTs). The rates of vital sign–related P-AEs and arterial blood gas analysis–related P-AEs were 57.1 % (252 IHTs) and 46.9 % (207 IHTs), respectively. Other P-AEs comprised mainly anxiety (66 IHTs, 15.0 %), agitation (49 IHTs, 11.1 %), resistance to ventilation when intubated (54 IHTs, 12.2 %), and pain or discomfort (27 IHTs, 6.1 %). The incidence of critical P-AEs was 33.1 % (146 IHTs) (Table 5). The rates of vital sign–related and arterial blood gas analysis–related critical P-AEs were 22.9 % (101 IHTs) and 15.0 % (66 IHTs), respectively. The majority of these AEs involved RR abnormality or more severe (54 IHTs, 12.2 %), HR abnormality or more severe (31 IHTs, 7.0 %), PaO_2_ abnormality or more severe (30 IHTs, 6.8 %), and lactate level abnormality or more severe (28 IHTs, 6.4 %). One accidental extubation and one airway obstruction occurred. No patient experienced cardiac arrest during the study period.

**Table 4:** Adverse events during intra-hospital transports (*N* = 441 intra-hospital transports evaluated)

Adverse event	Transports^a^ (*n*)	Transports (%)
Total AEs	352	79.8
Equipment- or staff-related AEs	35	7.9
Loss of monitor power	11	2.5
Vascular tubing obstructed	4	0.9
Disconnected or depleted of oxygen supply	9	2.0
Loss of ventilator power	3	0.7
Unexpected delay ≥15 minutes	8	1.8
Other	4	0.9
Patient-related AEs (P-AEs)	349	79.4
Vital sign–related P-AEs^b^	252	57.1
RR abnormality or more severe	99	22.5
HR abnormality or more severe	69	15.7
SBP abnormality or more severe	78	17.7
SpO_2_ abnormality or more severe	102	23.1
Arterial blood gas analysis–related P-AEs^b^	207	46.9
pH abnormality or more severe	53	12.0
PaO_2_ abnormality or more severe	70	15.9
PaCO_2_ abnormality or more severe	63	14.3
HCO_3_^−^ abnormality or more severe	36	8.2
Lactate level abnormality or more severe	42	9.5
Glucose level abnormality or more severe	39	8.8
New-onset arrhythmia	3	0.7
Anxiety	66	15.0
Agitation	49	11.1
Pain or discomfort	27	6.1
Resistance to ventilation when intubated	54	12.2
Accidental extubation	1	0.2
Nausea or vomiting	2	0.5
Airway obstruction	1	0.2
Other	2	0.5

*AE* adverse event, *RR* respiratory rate, *HR* heart rate, *SBP* systolic blood pressure, *SpO*_*2*_ pulse oximetry, *PaO*_*2*_ partial pressure of oxygen in arterial blood, *PaCO*_*2*_ partial pressure of carbon dioxide in arterial blood, *HCO*_*3*_^*−*^ bicarbonate level

^a^Number of transports with at least one AE

^b^Defined as an AE associated with detection of abnormal or more severe monitored parameters during the intra-hospital transport (IHT) period and post-IHT period

**Table 5:** Critical patient-related adverse events during intra-hospital transports (*N* = 441 intra-hospital transports evaluated)

Critical patient-related adverse events	Transports^a^ (*n*)	Transports (%)
Total critical P-AEs	146	33.1
Vital sign–related critical P-AEs^b^	101	22.9
RR abnormality or more severe	54	12.2
HR abnormality or more severe	31	7.0
SBP abnormality or more severe	21	4.7
SpO_2_ abnormality or more severe	11	2.5
Arterial blood gas analysis–related critical P-AEs^b^	66	15.0
pH abnormality or more severe	2	0.5
PaO_2_ abnormality or more severe	30	6.8
PaCO_2_ abnormality or more severe	7	1.6
HCO_3_^−^ abnormality or more severe	3	0.7
Lactate level abnormality or more severe	28	6.4
Glucose level abnormality or more severe	6	1.4
Accidental extubation	1	0.2
Airway obstruction	1	0.2

*P-AE* patient-related adverse events, *RR* respiratory rate, *HR* heart rate, *SBP* systolic blood pressure, *SpO*_*2*_ pulse oximetry, *PaO*_*2*_ partial pressure of oxygen in arterial blood, *PaCO*_*2*_ partial pressure of carbon dioxide in arterial blood, *HCO*_*3*_^*−*^ bicarbonate level

^a^Number of transports with at least one AE

^b^Defined as a parameter with more severe abnormality detected (category 2 or worse in Fig. 2)

When a vital sign–related P-AE was defined as an AE associated with detection of abnormal or more severe monitored parameters only in the post-IHT period, the rates of vital sign–related P-AEs and critical P-AEs were 29.5 % (130 IHTs) and 11.8 % (52 IHTs), respectively.

### Risk factors for P-AEs during IHT

Univariate and stepwise multivariate logistic regression analyses were performed to identify factors present before or during IHT that were related to an increase in P-AEs in critically ill patients during transport. Comparisons were made between reference category and each of the remaining groups per characteristic.

Table 6 shows the risk factors for P-AEs during IHT. Patient characteristics did not significantly affect the occurrence of P-AEs during transport. Univariate logistic regression analysis showed that the APACHE II score and vasoactive drug support before transport were associated with P-AEs during IHT. More specifically, patients with an APACHE II score ≥20 had a significantly higher incidence of P-AEs than did those with an APACHE II score ≤11 (*P* = 0.02). More P-AEs occurred in patients with a pH, PaCO_2_, lactate level, glucose level, HR, and RR in severity category 1 or 2 than in severity category 0 (*P* < 0.05). However, after adjusting for potential confounding factors through the multivariate analysis, only pH, PaCO_2_, lactate level, glucose level, and HR were independent influential factors for P-AEs during IHT (*P* < 0.05). Significantly more P-AEs occurred in patients with these parameters in severity category 1 or 2 than in severity category 0 (*P* < 0.05). Ventilation and transport characteristics were not associated with P-AEs during IHT. There was no evidence that patients receiving analgesia or sedation had more P-AEs during transport.

**Table 6:** Risk factors for patient-related adverse events during intra-hospital transport

Variable	Univariate analysis		Multivariate analysis	
	OR (95 % CI)	*P* value	OR (95 % CI)	*P* value
Patient characteristics				
Age, yr	1.00 (0.99–1.02)	0.67	NT	
Sex	0.94 (0.58–1.51)	0.79	NT	
Weight, kg	1.02 (1.00–1.04)	0.06	NT	
< 65	Reference	–		
≥ 65	1.46 (0.92–2.32)	0.11		
ICU admission type	1.04 (0.80–1.34)	0.79	NT	
Clinical characteristics before transport				
APACHE II score	1.43 (1.06–1.91)	0.02*		
≤ 11	Reference	–	Reference	–
12–19	1.25 (0.74–2.12)	0.40	1.27 (0.73–2.22)	0.40
≥ 20	2.11 (1.14–3.88)	0.02*	1.89 (0.98–3.67)	0.06
Glasgow Coma Scale score	1.14 (0.86–1.51)	0.36	NT	
15	Reference	–		
9–14	0.86 (0.47–1.56)	0.62		
≤ 8	1.39 (0.77–2.51)	0.27		
Artificial airway	1.26 (0.80–2.01)	0.32	NT	
Ventilation	1.22 (0.77–1.94)	0.40	NT	
Number of catheters	1.24 (1.00–1.54)	0.05	NT	
Arterial blood gas analysis				
pH				
7.35–7.45	Reference	–	Reference	–
< 7.35 or >7.45	1.55 (1.34–1.88)	0.01*	1.53 (1.32–1.88)	0.01*
PaO_2_	1.00 (0.99–1.00)	0.07	NT	
PaCO_2_, mmHg				
35–45	Reference	–		
< 35 or >45	1.61 (1.38–1.97)	0.04*	1.49 (1.29–1.81)	0.00*
Bicarbonate level, mmol/L			NT	
22–27	Reference	–		
< 22 or >27	1.08 (0.68–1.71)	0.75		
Lactate level, mmol/L	1.60 (1.17–2.18)	0.00*		
< 2	reference	–	Reference	–
≥ 2	2.11 (1.10–4.07)	0.03*	1.47 (1.04–2.08)	0.03*
Glucose level, mmol/L				
4.0–10.0	Reference	–		
< 4 or >10	2.27 (1.21–4.28)	0.01*	1.97 (1.01–3.84)	0.04*
Vital signs				
SBP, mmHg			NT	
90–150	Reference	–		
< 90 or >150	1.37 (0.67–2.82)	0.39		
DBP, mmHg	1.01 (1.00–1.03)	0.12	NT	
MAP, mmHg	1.02 (1.00–1.03)	0.08	NT	
Heart rate				
50–110	Reference	–	Reference	–
< 50 or >110	3.02 (1.40–6.51)	0.00*	2.73 (1.21–6.16)	0.02*
Respiratory rate				
12–25	Reference	–	Reference	–
< 12 or >25	2.33 (1.19–4.59)	0.01*	2.00 (0.98–4.10)	0.06
Pulse oximetry	0.93 (0.85–1.02)	0.13	NT	
Analgesia	1.60 (0.78–3.27)	0.20	NT	
Sedation	1.42 (0.80–2.54)	0.24	NT	
Vasoactive drug support	2.02 (1.09–3.75)	0.03*	1.85 (0.97–3.55)	0.06
Transport characteristics				
Analgesia	1.25 (0.56–2.78)	0.59	NT	
Sedation	1.07 (0.58–1.95)	0.83	NT	
Vasoactive drug support	1.60 (0.86–2.99)	0.14	NT	
Emergency transport	0.52 (0.26–1.03)	0.06	NT	
Multiple IHTs of one patient	1.29 (0.76–2.18)	0.34	NT	
Night transport	0.64 (0.22–1.91)	0.43	NT	
Transport duration, minutes	1.01 (0.99–1.03)	0.32	NT	

*APACHE* Acute Physiology and Chronic Health Evaluation, *PaO*_*2*_ partial pressure of oxygen in arterial blood, *PaCO*_*2*_ partial pressure of carbon dioxide in arterial blood, *SBP* systolic blood pressure, *DBP* diastolic blood pressure, *MAP* mean arterial pressure, *NT* not tested, *OR* odds ratio, *CI* confidence interval

*P < 0.05

Risk factors for critical P-AEs during IHT are shown in Table 7. Univariate logistic regression analysis identified pre-IHT parameters or transport characteristics associated with P-AEs during IHT (*P* < 0.05), namely weight, APACHE II score, GCS score, number of catheters, PaO_2_, lactate level, glucose level, HR, RR, SpO_2_, sedation before transport, vasoactive drug support during transport, and emergency transport. In the multivariate logistic regression analysis, APACHE II score, PaO_2_, lactate level, glucose level, HR, RR, SpO_2_, and sedation before transport were independent influential factors for critical P-AEs during IHT (*P* < 0.05). Furthermore, patients with an APACHE II score ≥20 had a significantly higher incidence of critical P-AEs than did patients with an APACHE II score ≤11 (*P* = 0.01). A significantly higher rate of critical P-AEs occurred in patients with a parameter pre-IHT (PaO_2_, lactate level, glucose level, HR, RR, and SpO_2_) in severity category 1 or 2 than in severity category 0 (*P* < 0.05). Ventilation, night transport, multiple IHTs of one patient, and transport duration were not associated with critical P-AEs during IHT.

**Table 7:** Risk factors for critical patient-related adverse events during intra-hospital transport

Variable	Univariate analysis		Multivariate analysis	
	OR (95 % CI)	*P* value	OR (95 % CI)	*P* value
Patient characteristics				
Age, yr	1.00 (0.99–1.01)	0.71	NT	
Sex	0.70 (0.46–1.08)	0.10	NT	
Weight, kg	1.03 (1.01–1.04)	0.00*		
< 65	Reference	–	Reference	–
≥ 65	1.95 (1.30–2.94)	0.00*	1.55 (0.95–2.54)	0.08
ICU admission type	0.95 (0.76–1.19)	0.67	NT	
Clinical characteristics before transport				
APACHE II score	1.66 (1.29–2.14)	0.00*		
≤ 11	Reference	–	Reference	–
12–19	1.66 (1.01–2.74)	0.04*	1.44 (0.79–2.63)	0.23
≥ 20	2.75 (1.66–4.57)	0.00*	2.49 (1.23–5.03)	0.01*
Glasgow Coma Scale score	0.76 (0.60–0.96)	0.02*		
15	Reference	–	Reference	–
9–14	0.93 (0.53–1.64)	0.80	0.67 (0.33–1.38)	0.28
≤ 8	1.82 (1.14–2.91)	0.01*	0.89 (0.46–1.74)	0.74
Artificial airway	1.38 (0.92–2.07)	0.12	NT	
Ventilation	1.48 (0.99–2.20)	0.06	NT	
Number of catheter	1.28 (1.07–1.54)	0.01*	1.20 (0.95–1.51)	0.13
Arterial blood gas analysis				
pH			NT	
7.35–7.45	Reference	–		
< 7.35 or >7.45	1.03 (0.69–1.54)	0.88		
PaO_2_, mmHg	0.99 (0.99–1.00)	0.00*		
≥ 80	Reference	–	Reference	–
< 80	2.49 (1.61–3.86)	0.00*	2.26 (1.31–3.91)	0.00*
PaCO_2_, mmHg			NT	
35–45	Reference	–		
< 35 or >45	1.36 (0.91–2.02)	0.13		
Bicarbonate level, mmol/L			NT	
22–27	Reference	–		
< 22 or >27	1.28 (0.86–1.92)	0.23		
Lactate level, mmol/L	1.87 (1.51–2.33)	0.00*		
< 2	Reference	–		
≥ 2	2.82 (1.77–4.49)	0.00*	3.12 (1.75–5.58)	0.00*
Glucose level, mmol/L				
4.0–10.0	Reference	–		
< 4 or >10	2.24 (1.43–3.50)	0.00*	1.80 (1.05–3.08)	0.03*
Vital signs				
SBP, mmHg			NT	
90–150	Reference	–		
< 90 or >150	1.10 (0.62–1.95)	0.74		
DBP, mmHg	1.00 (0.98–1.01)	0.64	NT	
MAP, mmHg	1.00 (0.99–1.02)	0.95	NT	
Heart rate				
50–110	Reference	–		
< 50 or >110	4.53 (2.76–7.42)	0.00*	2.97 (1.66–5.32)	0.00*
Respiratory rate				
12–25	Reference	–		
< 12 or >25	3.06 (1.92–4.89)	0.00*	2.45 (1.39–4.33)	0.00*
Pulse oximetry	0.84 (0.78–0.91)	0.00*		
≥ 95	Reference	–		
< 95	3.56 (1.94–6.52)	0.00*	2.79 (1.31–5.95)	0.01*
Analgesia	1.34 (0.78–2.30)	0.28	NT	
Sedation	1.96 (1.25–3.09)	0.00*	1.87 (1.07–3.29)	0.03*
Vasoactive drug support	1.51 (0.96–2.37)	0.07	NT	
Transport characteristics				
Analgesia	0.90 (0.47–1.76)	0.76	NT	
Sedation	1.27 (0.77–2.10)	0.36	NT	
Vasoactive drug support	1.76 (1.10–2.83)	0.02*	1.63 (0.92–2.88)	0.10
Emergency transport	1.54 (1.33–1.89)	0.01*	1.26 (0.68–2.35)	0.47
Multiple IHTs of one patient	0.84 (0.54–1.31)	0.45	NT	
Night transport	0.83 (0.37–1.87)	0.65	NT	
Transport duration, minutes	0.99 (0.97–1.01)	0.18	NT	

*APACHE* Acute Physiology and Chronic Health Evaluation, *PaO*_*2*_ partial pressure of oxygen in arterial blood, *PaCO*_*2*_ partial pressure of carbon dioxide in arterial blood, *SBP* Systolic blood pressure, *DBP* diastolic blood pressure, *MAP* mean arterial pressure, *NT* not tested, *OR* odds ratio, *CI* confidence interval

*P < 0.05

## Discussion

To our knowledge, this is the first prospective multicenter study of the incidence and risk factors for AEs during IHT in both medical and surgical ICU patients, including both mechanically ventilated and non-ventilated patients. Three previous reports of IHT of ICU patients contained more transports than our study. One observational study was carried out in a cohort of 452 IHTs of 226 adults and infants from 3 anesthesiology ICUs in Austria [6]. In total, 4.2 % of IHTs were associated with a critical incident. Kue et al. [5] reported an AE rate of 1.7 % in a retrospective study of 3358 IHTs by a specialized transport team in the United States. AEs included hypoxia and alterations in blood pressure. Furthermore, a multicenter cohort of 1782 mechanically ventilated adult ICU patients with 3006 IHTs experienced 621 AEs (37.4 %). The authors of that study compared 1659 transported patients with 3344 patients who were not transported [2]. Pneumothorax, atelectasis, ventilator-associated pneumonia, hypoglycemia, hyperglycemia, and hypernatremia were reported as complications that occurred more frequently in the transported population. A longer ICU stay, but not a higher mortality rate, was found in transported patients than in non-transported patients. No risk factors for AEs were reported. Perhaps an approach integrating multiple vital signs derangements in one score, such as the Modified Early Warning Score, might be helpful as a predictor. Additionally, AEs have been reported in many studies with more critically ill patients during inter-hospital transport [18–23] than we included in our study, but few data have documented the risks [24–26]. Although these findings were derived from studies on inter-hospital transport, they may also apply to IHT [20].

### Definition of AEs during IHT

Reported rates of transfer-related AEs vary among different studies, not only because of differences in incidences but also because different definitions were used [22]. How to more reasonably define vital sign–related or laboratory work–related AEs is unclear. First, conditions of critically ill patients are prone to change even without transport. It is difficult to tell if these changes would have occurred if the patients had remained where they were. Second, no definition perfectly distinguishes whether such changes are actually adverse or simply represent physiologic variability among patients. Third, sicker patients are more likely to deteriorate during transfer [23]. Therefore, even a minimal change in vital signs or laboratory work might be clinically important.

Vital signs and arterial blood gas analysis findings were classified according to their severity in our study, and changes in severity categories were used to define vital sign–related and arterial blood gas analysis–related P-AEs. However, not all of the above-mentioned problems can be solved by this definition. We believe that a consensus on the definition of transfer-related AEs must be reached in the future to allow for appropriate comparison of AE rates.

### Incidence of AE

In this study, we evaluated 369 adult critically ill patients with 441 IHTs. The overall incidence of AEs was 79.8 %, and 33.1 % of IHTs were associated with a critical P-AE. These findings are similar to those in some previous reports [9, 12–14] but greater than those in the largest studies evaluating IHT of ICU patients. These results are noteworthy, such that physicians should pay greater attention to the safety of critically ill patients during IHT.

A greater number of clinical characteristics were assessed in our study than in previous reports. The high AE rate found in our study is attributable to the definition of P-AE. First, vital signs were monitored and noted every 5 minutes during transport; thus, more transient events might have been captured than in previous studies [2]. In total, 57.1 % of the IHTs were associated with vital sign–related P-AEs, and 22.9 % of the IHTs were associated with vital sign–related critical P-AEs. However, if changes in these variables during the IHT period were not detected (vital signs were observed or collected just before and after transport, like in most previous studies), the rates of vital sign–related P-AEs and critical P-AEs were 29.5 % and 11.8 %, respectively. More than half of the vital sign–related P-AEs might not have been identified. Second, the careful observation of arterial blood gas values before and after IHT may also have contributed to the high AE rate, assessed in few previous studies [12, 27]. The incidence of arterial blood gas analysis–related P-AEs was unexpectedly high (46.9 % of IHTs). Additionally, AEs during IHT related to lactate (42 IHTs, 9.5 %) and bicarbonate (36 IHTs, 8.2 %) levels have not been reported previously. Patient glucose levels during IHT were described in only one recent report [2]. The rates of hypoglycemia and hyperglycemia in this study were 3.38 % and 23.75 %, respectively. The percentage of glucose level deterioration was 8.8 % (39 IHTs) in our study, and a low rate of critical glucose level deterioration was found in our patients (6 IHTs, 1.4 %). These occurrences may be secondary to interruptions in nutrition support or insulin therapy during transport.

Identification of E-AEs is important because such AEs can lead to P-AEs. The incidence of E-AEs in the present study (35 IHTs, 7.9 %) was lower than rates reported in previous studies (11–34 %) [13, 28]. Most E-AEs were related to a disconnected power source or power failure of a monitor, a disconnected or depleted oxygen supply, or unexpected delays. These AEs were associated with insufficient preparation before IHT that did not take into account potential risk factors. Careful preparation of equipment before transport and assistance by well-trained personnel during IHT should minimize these problems.

### Risk factors for P-AEs

Accurate assessment of the risk/benefit ratio of each transport is the key to reducing AEs during IHT of critically ill patients. Physicians should consider both the indications for and risk factors associated with IHT.

Adult ICU assessment models of illness severity have been used to predict patient outcomes for three decades [29, 30]. Several researchers have evaluated the predictive value of AEs during transport of critically ill patients. The Therapeutic Intervention Scoring System score is reportedly associated with the occurrence of AEs during transport [31]. The Simplified Acute Physiology Score II and Sequential Organ Failure Assessment score have not been reported to predict AEs during transport [3, 25]. Disease severity as assessed by APACHE II scores was correlated with a higher risk of physiologic deterioration [6]. In the present study, the APACHE II score on the day of IHT was not an independent influential factor for P-AEs during IHT, but patients with an APACHE II score ≥20 had a significantly higher incidence of critical P-AEs than did patients with an APACHE II score ≤11. Further work is needed to evaluate the use of illness severity scores in predicting AEs during IHT.

The use of artificial airways was not associated with P-AEs during IHT by logistic regression analysis. Ventilated patients experience physical discomfort, anxiety, and hemodynamic instability and might be at higher risk for P-AEs during transport. However, our study showed that mechanical ventilation was not a risk factor for P-AEs during IHT, in contrast to previous reports [6, 26]. This result may be because of the lower incidence of E-AEs in our study or because ventilation settings were not analyzed in this study. Better characterization of these risks in patients requiring ventilation is needed.

Excluding the bicarbonate level, arterial blood gas analysis parameters before transport were significantly related to P-AEs or critical P-AEs during IHT in our study. These variables have not been studied as potential risk factors for AEs during IHT of ICU patients. Strauch et al. [25] reported that patients who died within 24 h after inter-hospital transport had a lower pH. Patients with an abnormal pH and PaCO_2_ were more prone to developing P-AEs during transport, and PaO_2_ was an independent influential factor for critical P-AEs. Blood lactate concentrations have been widely used as an indicator of disease severity [32, 33], and elevated lactate clearance is reportedly predictive of lower mortality in critically ill patients [34]. A high lactate level (≥2 mmol/L) before transport was a predictive factor for P-AEs or critical P-AEs during IHT of the patients in the present study. Additionally, a glucose level <4 or >10 mmol/L before transport was identified as a predictive factor for P-AEs or critical P-AEs during IHT.

Patients with physiologic instability before transport had a higher incidence of AEs during inter-facility transport [18]. Abnormal vital signs are reported to be strongly associated with adverse outcomes [15]. Our study found that vital signs were associated with P-AEs during IHT; this has not been described in previous studies. Patients with an HR <50 or >110 had more P-AEs than those in a safe severity category. Furthermore, RR and SpO_2_ were both influential factors for critical P-AEs during IHT. However, blood pressure was not found to be related to the occurrence of P-AEs in our study.

The duration of transport is also reportedly associated with AEs during transport of critically ill patients [26, 31]. IHT should be coordinated with the destination to shorten travel time and delays as much as possible. However, our results showed that a long IHT duration was not a significant risk factor for P-AEs, which is the same result found by Seymour et al. [24]. Unlike a similar study [6], emergency transport was not found to significantly increase the risk of P-AEs in our patients. Night transport and multiple IHTs of the same patient at different times were not risk factors for P-AEs during IHT, as previously described [3, 6]. These transport-related factors must be further investigated with more IHT cases.

Anxiety or agitation was recorded in more than 25 % of transports. Pain, discomfort, and resistance to ventilation when intubated occurred in about 19 % of IHTs. These AEs were perhaps due to inadequate analgesia and sedation in our patients. Patient sedation before transport is a well-described risk factor for AEs during IHT [3], but sedation was associated only with critical P-AEs in our study. Further research on this association is needed.

### Strategies to minimize AEs

Various maneuvers have been reported to improve patient IHT outcomes. Transport monitors are not routinely used during the IHT of ICU patients in China, although recommendations for their use have been published [35]. Transport monitoring is an essential part of IHT. Manual ventilation of critically ill mechanically ventilated patients can be performed safely during transport [36]. However, transport ventilators provide more reliable and stable ventilatory support than do manual ventilators and are preferable for IHT [27]. Non-invasive positive pressure ventilation is increasingly being used in patients with acute respiratory failure in the ICU setting [37]. The authors of one study reported that dedicated non-invasive ventilators allow better patient–ventilator synchrony than do ICU and transport ventilators [38]. Availability of a medical emergency team can also improve outcomes after AEs [39]. Use of resistive heating has been shown to be effective in maintaining the core temperature of ICU patients during IHT [40].

Recommendations for the IHT of critically ill patients have been published [4, 35, 41–44]. These recommendations cover pretransport coordination and communication, care of patient equipment, patient monitoring during transport, preparation of the patient before transport, documentation of transport, and training of caregivers involved in the transport processes. However, hospitals should implement policies and procedures to mitigate the risks associated with IHT in practice [45].

### Study limitations

This study has several limitations. The impact of IHT on patient outcomes, such as the ICU or hospital length of stay and mortality rate, was not evaluated. Risk factors for E-AEs during IHT were not analyzed, owing to the inadequate case numbers. Because more than 50 % of the data for pain scales and sedation scores before or during transport were missing, their predictive values for AEs were not assessed in our study. Patient diagnosis, electrolyte levels, use of neuromuscular blockade medication, use of nutritional support, fluid therapy, use of infusion pumps, ventilator modes and settings, and accompanying personnel were also not evaluated as potential influential factors for P-AEs. Finally, standardized methods to transport patients, such as the use of transport protocols or well-trained transport teams, may reduce the incidence of AEs during transport. These programs used in the ICUs that participated in our study might have been important influential factors for AEs. However, the information was not recorded, and further research is needed.

## Conclusions

To our knowledge, this is the first prospective multicenter study to comprehensively identify the incidence and risk factors of AEs during the IHT of different ICU patients. A high P-AE rate was found in our patients. Risk factors for P-AEs during IHT were identified. These included abnormal pH and PaCO_2_, high lactate levels, and specific glucose levels before transport. Critical P-AEs were associated with the APACHE II score, PaO_2_, lactate level, glucose level, HR, RR, SpO_2_, and sedation before transport. Strategies designed to minimize AEs during IHT are needed in practice.

## Key messages

- The incidence of P-AEs during IHT of ICU patients in this multicenter study in China was very high.
- New risk factors for P-AEs during IHT were abnormal PH and PaCO_2_, high lactate levels, and specific glucose level before transport. Critical P-AE was associated with APACHE II score, PaO_2_, lactate level, glucose level, HR, RR, SpO_2_, and sedation before transport.
- Strategies designed to minimize AE during IHT are needed in practice.

## Abbreviations

ABG: arterial blood gas
AE: adverse event
APACHE: Acute Physiology and Chronic Health Evaluation
CI: confidence interval
DBP: diastolic blood pressure
E-AE: equipment- and staff-related adverse event
GCS: Glasgow Coma Scale
HCO_3_^−^: bicarbonate level
HR: heart rate
ICU: intensive care unit
IHT: intra-hospital transport
MAP: mean arterial pressure
MEWS: Modified Early Warning Score
NT: not tested
OR: odds ratio
PaCO_2_: partial pressure of carbon dioxide in arterial blood
P-AE: patient-related adverse event
PaO_2_: partial pressure of oxygen in arterial blood
RR: respiratory rate
SBP: systolic blood pressure
SpO_2_: pulse oximetry
